# Green innovation and environmental quality in OECD countries: the mediating role of renewable energy and carbon taxes

**DOI:** 10.1007/s11356-023-31111-5

**Published:** 2023-12-06

**Authors:** Kafeel Kafeel, Jing Zhou, Monmala Phetkhammai, Lu Heyan, Sher Khan

**Affiliations:** 1 Business School, Xiangtan University, Xiangtan, Hunan China; 2 Faculty of Management, Department of International Management, Comenius University Bratislava, Bratislava, Slovakia

**Keywords:** CO2 emissions, Eco-innovation, Environmental tax, Renewable energy

## Abstract

The exceptional rise in overall economic activities has deteriorated environmental sustainability around the world. However, countries around the globe are implementing strategies for reaching the global climate objective. For this purpose, OECD countries committed many efforts, although their pledges and results are not parallel to the level of the Paris Agreement’s ambition. This study examines the impact of eco-innovation, environmental taxes, and renewable energy consumption on the environmental performance of selected OECD countries over the period of 2006 to 2020. This study uses the generalized method of moments (GMM) and instrumental variables 2 stage least square (2SLS) methods. For robustness checks, this study uses a quantile regression approach. We conclude that an increase in the adoption of renewable energy and green innovation has a statistically significant impact on controlling CO2 emissions. Moreover, the empirical model is expanded by incorporating environmental taxes as an explanatory variable. The expanded model showed that the imposition of environmental taxes has a detrimental impact on the reduction of CO2 emissions. Moreover, on the contrary, an increase in economic activities, measured by GDP, is responsible for rising CO2 emissions in OECD countries. In light of the results we obtained, policy recommendations are provided.

## Introduction

A worldwide appeal for sustainable solutions to minimize greenhouse gas emissions (GHG) and alleviate the adverse effects of carbon dioxide (CO2) on the environment has been sparked by the rising climate change hazards. One of the possible reasons for environmental degradation is the threat of global natural resource exhaustion required for economic activities (Munasinghe 1998). Thus, during 1990–2008, the world’s CO2 emissions rose from 22.5bn tons to 31.5bn tons, which is a severe challenge for sustainable growth and the environment (OECD 2021). For this purpose, the recently held COP-26 conference for the implementation of the Paris Agreement and Kyoto Protocol to engage their member countries to cope with environmental-related problems. Emissions of Carbon dioxide have been at a declining rate in the case of OECD countries due to their strengthened climate policies and the post-financial crises of 2008. However, their progress is still lacking, and they are expected to confront a rise in CO2 emissions due to the increased demand for energy use and CO2-related emissions. On average, the largest emitter of GHG emissions is the energy sector, which creates about 29% of GHG emissions in the entire OECD countries, tailed by the transportation sector (13%), agriculture sector (09%), and industrial sector (07%). Given the disastrous climate change due to increased economic activities through higher energy demand in industrialization, countries are devising their strategies to control emissions of greenhouse gases. These strategies include eco-innovation, green tax policies, carbon pricing, and the adoption of renewable energy. Environmental innovation is one of the key forces in the effort to create a low-carbon economy. Environmental innovation refers to a broad spectrum of scientific developments, legal frameworks, and commercial strategies that are intended to reduce negative environmental effects, improve resource efficiency, and support sustainable development. Environmental innovation positively influences the reduction of CO2 emissions through developments in clean technology, renewable energy, and energy efficiency (Costantini et al. 2017; Righi et al. 2016). The adoption of sustainable practices to address the carbon emissions issue can be facilitated by policies and laws that encourage R&D investment and offer financial incentives for green technologies. For example, the Commission of the EU for Emissions Trading System & Renewable Energy Directive has been crucial in fostering environmental innovation and reaching emission reduction goals (Bleischwitz et al. 2018). Additionally, the circular economy idea places an emphasis on resource efficiency, recycling, and reuse, which reduces waste and emissions over the course of a product’s existence. Therefore, to have a beneficial effect on environmental sustainability, eco-innovation extends beyond technology improvements and includes modifications to business models and consumer behavior.

As the world struggles to address the urgent need to halt climate change, environmental taxes have grown to be a powerful tool for policymakers. Environmentally related tax is also used as a device that aims to prevent actions that raise GHG emissions and reduce carbon footprints by levying fees on polluting activities. Modern economies internalize pollution’s external costs and encourage environmentally beneficial behavior. Parry et al. (2016) asserted that CO2 emissions in the US states of Walls and Harrington were lowered by 2–3% as a result of the 10% gasoline tax. In the past, environmental tax policies implemented by many countries have proved to have a desirable impact on environmental sustainability. For example, the carbon tax policy in 1991 in Norway, the comprehensive energy system and CO2 tax system in Sweden, the vehicle-related tax in Finland and Energy, and CO2 tax in Denmark have shown favorable impacts on controlling emissions of carbon dioxide (Eyckmans and Hagem 2011; Baldursson et al. 2022; Toppinen et al. 2019). In the case of OECD countries, the climate tax as a percentage of total tax revenue has been increased over the years.

Fossil fuel energy is the largest generator of GHG emissions. OECD countries still have reliance on fossil fuels energy, which accounts for about 80% of their energy consumption (OECD 2021). To combat climate change and cut down on emissions of greenhouse gases, switching to renewable energy sources is an appealing option. Solar, wind, hydro, and biomass are examples of renewable energy sources that promise to replace fossil fuel-based energy output and reduce carbon dioxide (CO2) emissions. For instance, Wang et al. (2017) discovered that in China, a 0.036 percentage point drop in carbon dioxide emissions is achieved for every 1% increase in renewable energy utilization. Similarly, according to research by Waris et al. (2023), using more solar energy and biofuel fuels can significantly lower CO2 emissions in G20 countries. According to the Inter-governmental Panel on Climate Change (IPCC), global warming must stop at 1.5°C by 2050. In addition, the International Energy Agency (IEA) also emphasizes the significance of renewable energy in reaching global climate targets. The World Energy Outlook 2020 report underlines the importance of raising the share of sources of renewable energy in the energy mix in order to come up with the mission of net-zero emissions by the year 2050 (IEA 2021a, b).

This study intends to analyze the impact of eco-innovation in the presence of environmental taxes (ETAX) and renewable energy consumption (REC) on carbon emissions as the case study of 21 OECD economies over the periods of 2006–2020. The basic purpose of this research is to devise strategies to abate CO2 emissions in the 21 OECD countries (see Table 6 for a list of countries). This study addresses several pressing problems. The study analyzes the effectiveness of eco-innovation policies, a critical issue for policymakers seeking sustainable growth. The contribution of this research lies in empirically demonstrating whether eco-innovation policies indeed lead to significant emissions reductions, thereby providing evidence-based guidance for policy formulation. Moreover, it seeks to quantify the impact of environmental taxes, shedding light on the effectiveness of market-based mechanisms in incentivizing emissions reductions, a contribution that can inform the design of future environmental tax policies. Furthermore, the examination of the role of renewable energy consumption in emissions mitigation contributes by elucidating the extent to which transitioning to renewable sources aligns with emissions reduction goals. Novelty emerges from the nuanced insights that can be gained, such as identifying optimal tax levels and conditions for renewable energy effectiveness, thus advancing our understanding of effective environmental policy design. When addressing pressing global issues, the significance of this study cannot be exaggerated. The adverse impacts of climate change, pollution, and biodiversity loss constitute significant threats to the well-being of both current and future generations. Consequently, the urgency to address environmental sustainability has become an issue of growing concern. This research provides valuable support to policymakers by elucidating the effectiveness of measures such as eco-innovation, environmental taxes (ETAX), and renewable energy consumption (REC) in enhancing environmental quality. This facilitates the formulation of legislation and incentives by policymakers that are well-informed and efficacious. Furthermore, it offers valuable insights to companies operating in the green technology and renewable energy sectors, enabling them to make informed decisions regarding their investment strategy and market positioning. Furthermore, this initiative enhances the existing leadership of OECD countries in the realm of sustainable development, therefore serving as an exemplar for other nations and fostering global collaboration in addressing shared environmental challenges. This research possesses the capacity to include individuals in the practice of environmental stewardship through the enhancement of public awareness and the promotion of environmental policies. This will foster a sense of civic engagement among individuals, prompting them to endorse policies aimed at addressing global issues. The research conducted in this field significantly contributes to the academic community, generating findings that can influence resource allocation and long-term sustainability planning. Consequently, it plays a crucial role in shaping a future characterized by enhanced resilience and environmental responsibility.

This paper is organized in different sections. In the “Literature review” section, reviews of the literature are discussed. The “Research methodology and data identification” section highlights the methodology and data description, and the result, discussion, and policy recommendations are discussed in the “Results and discussions” and “Conclusions and policy implications” sections, respectively.

## Literature review

Existing literature has attempted well to address the discussion of various measures to cope with the environmental degradation problem. Being focused on the objective of the research, this section extracts existing literature related to the role of eco-innovation (EI), environmental tax (ETAX), and REC on CO2 emissions.

### Review of the literature on eco-innovation and CO2 emissions

The relationship between EI and CO2 emissions remained the subject of extensive research (Qi et al. 2014; Costantini et al. 2017; Erdoğan et al. 2020; Shobande et al. 2023). Eco-innovation has been added to the empirical model by academics to more accurately analyze the factors that boost CO2 emissions. Erdoğan et al. (2020) assessed the contribution of environmental innovation to the reduction of CO2 emissions in the group of G20 economies. They found that using green technology in manufacturing reduced CO2 emissions. According to Zoundi (2017), the use of renewable energy, natural resource utilization, and technological advancements in the energy sector all contribute to a more sustainable world. The significance of eco-innovation in controlling CO2 emissions in European industry has been examined by Costantini et al. (2017), Shobande and Ogbeifun (2021), and Shobande and Asongu (2023). In their findings, EI is anticipated to help raise environmental standards. Similarly, Shahbaz et al. (2020) found that introducing energy innovations and energy-efficient technology can have an impact on environmental sustainability. Safi et al. (2022) underlined the importance and significance of eco-innovation in lowering carbon emissions in China. Similarly, Zhang (2023) stressed that eco-innovation can lower CO2 emissions and enhance environmental sustainability when combined with technological breakthroughs. Alvarez-Herranz et al. (2017) emphasizes the significance of low-carbon technologies in order to promote environmentally friendly growth in the group of seventeen OECD countries. According to the authors, using energy-efficient equipment would ease the switch from conventional energy to green and clean energy sources. As a result, the production of carbon dioxide is inversely correlated with eco-innovation. However, Robinson (2019) claimed that the adoption of some innovations, such as e-commerce and digital technology, may also result in higher CO2 emissions and energy consumption. It is crucial to understand that a variety of factors, such as market dynamics, governmental support, financial resources, and technological readiness, can affect how eco-innovation affects the decrease of carbon emissions (Geissdoerfer et al. 2017; Shobande 2021). In the study of Li et al. (2020), it is asserted that eco-innovation can have a substantial impact on environmental sustainability. Chien et al. (2021) promoted eco-innovation as a way to achieve zero carbon emissions. The author found that eco-innovation worked together with other measures to cut carbon dioxide emissions and increase efficiency. Similar findings were made by Wei and Li-hua (2022), who found that eco-innovations, particularly those in R&D, have a negative long-term impact on CO2 emissions. According to Hojnik and Ruzzier (2016), eco-innovation, especially in cleaner technology, can sustainably improve environmental quality. According to the findings of Ahmed et al.’s (2016), technological innovation helps to reduce carbon emissions in a few European countries (See Henriques and Borowiecki 2017 for similar results). Furthermore, Naz and Aslam (2023) emphasized that eco-innovation lowers CO2 emissions by improving and changing current goods and services. Naz and Aslam (2023) looked at the cyclical impact of tech innovation in the environmental sector on CO2 emissions in the South Asia. They found that positive shocks in the development of environmental technologies result in a drop in CO2 emissions. The association between CO2-reducing innovations and outsourcing in the sustainable buildings and photovoltaics industries was studied by Leoncini et al. (2016). Their findings showed that advances in CO2 reduction are positively impacted by outsourcing concrete operations. Additionally, Ma et al. (2022a) stressed that eco-innovation has a detrimental impact on CO2 emissions, showing that environmental technological innovation lowers consumption-based CO2 emissions. Thus, a study of the literature shows how eco-innovation has a major impact on lowering CO2 emissions. Eco-innovation is essential for reducing environmental pollution and advancing sustainable development through the use of cleaner technology, R&D expenditures, and process enhancements. We construct the given hypothesis.

### Literature on environmental taxes and CO2 emissions

There have been numerous research that study how environmental taxes affect emissions of CO2. Numerous studies have importance in the existing literature on this topic, and their findings have absorbed the correlation between environmental taxes and carbon dioxide (CO2) emissions, which have shed important light on the efficacy of such regulations. Economic policy uncertainty (EPU) and CO2 emissions in China were studied by Liu et al. (2021). According to their study, EPU directly affects CO2 emissions and indirectly affects them through environmental legislation. Ghazouani et al. (2020) conducted research on how different European countries’ carbon tax regimes affected their CO2 emissions. Their empirical findings revealed that environmental levies might not be successful in lowering pollution and carbon emissions. The effects of environmental taxes on carbon emissions in OECD economies were assessed by Bashir et al. in 2020. They found that carbon emissions are negatively impacted by environmental fees utilizing system-GMM and quintile regression methods. Ulucak et al. (2020) looked at how different carbon prices affected the economy and the environment. Their research suggests that a higher carbon tax rate at its foundation can lead to more money being put towards measures to reduce emissions, which in turn can lead to environmental sustainability. In the study of Wisdom et al. (2022), they focused on the need to manage carbon taxes well, especially in developing nations. They emphasized the paucity of studies examining how carbon prices affect Sub-Saharan Africa’s economic growth. According to Liu et al. (2021), the reduction of carbon emissions and improved environmental conditions are just a couple of the advantages of collecting carbon taxes. They also talked about the economic effects of carbon prices, emphasizing the possibility of a “double dividend” in accordance with the tax neutrality principle. Silajdzic and Mehic (2018a) investigated the functional relationship between energy consumption and transport taxes on the reduction of CO2 emissions in the countries under transition. They discovered that environmental levies could reduce CO2 emissions despite frequent worries about the negative effects on economic costs and business competitiveness. Mundaca et al. (2021) examined the consequences of carbon pricing on international transportation fuels, as well as its effects on carbon emissions and trade activity. Their research showed that rises in fuel prices, which may be viewed as a stand-in for fuel taxes, have a significant impact on the volume of maritime transport, the use of bunker fuel, and carbon emissions from the global shipping industry. Using environmental taxes as a percentage of overall tax revenue, Sommer and Hargrove (2020) looked at the connection between environmental governance and CO2 emissions for 75 different countries. According to their analysis, reduced CO2 emissions are related to better environmental governance, which is reflected in higher environmental levies. The impact of energy and carbon taxes on household welfare and CO2 emissions in Mexico was examined by Renner et al. (2018). They discovered that household welfare and carbon emissions could be affected by environmental taxes in both favorable and unfavorable ways. The complicated connection between REC and CO2 emissions and environmental legislation in China was examined by Zhang et al. (2022b). They emphasized the importance of considering the “green paradox” and other potential unintended consequences of environmental rules, such as the encouragement of fossil fuel mining and CO2 emissions. The irregular dynamics of oil prices and environmental degradation, including CO2 emissions, in Pakistan were investigated by Qudrat-Ullah (2022). While they did identify a correlation between oil prices and environmental deterioration, they also found that the usage of renewable energy and GDP were more significant in influencing CO2 emissions. Li and Zhao (2017) investigated the policy impact of the environment of carbon pricing with a particular emphasis on the situation in Sweden. They discovered that Sweden’s carbon tax helped to reduce CO2 emissions, demonstrating the potency of carbon taxation as a tool for environmental policy. The effectiveness of environmental policy was examined by Nerudova et al. (2016), along with the effect of environmental tax ratios on CO2 emissions. They discovered a negative relationship between tax rates and CO2 emissions, indicating that higher tax rates may help reduce emissions.

### Literature review on renewable energy consumption and CO2 emissions

The utilization of renewable energy as a means of combating climate change and lowering emissions of greenhouse gases like carbon dioxide (CO2) has received considerable attention in recent years. Examining the actual evidence and major conclusions from prior studies, this literature review strives to provide a thorough knowledge of the connection between renewable energy and carbon emissions. Mensah et al. (2018) discovered that employing renewable energy sources could reduce carbon emissions in 28 tested OECD economies. Inglesi-Lotz and Dogan (2018) also support the claim that REC is better than non-renewable energy sources for reducing CO2 emissions. The results of the study by Hu et al. (2018) give additional support for the use of REC for CO2 emissions control in the case of 25 chosen emerging economies (see Bölük and Mert 2015 for similar results). According to Alvarez-Herranz et al. (2017), in addition to eco-innovation, green energy is helpful in dropping CO2 emissions in 17 OECD countries. In a meta-analysis of 128 papers, Lu et al. (2019) discovered a consistent inverse association between REC and CO2 emissions. The results showed that across nations and industries, increasing the use of REC results in a significant decrease in CO2 emissions. Similar findings were found by Zheng et al. (2020), who looked at how renewable energy in China affected CO2 emissions. The functional relationship between the production of renewable energy and CO2 emissions in the member states of the European Union (EU) was examined by Soares et al. (2018). Their results showed that REC is beneficial in lowering the carbon intensity of the electrical industry. Similarly, the impact of using renewable sources on lowering carbon emissions in G7 countries was investigated by Zafar et al. (2019). They discovered an inverse relationship between the two factors. A study conducted by Li et al. (2020) examined the efficiency and trade perspectives of improving energy resource quality and reducing CO2 emissions in China and Nigeria. Moreover, Zhu et al. (2020) discovered that increasing the use of renewable energies resulted in a sizable decrease in CO2 emissions in China. They argued that government support is necessary in addition to private investment, which is essential but insufficient for promoting eco-friendly energy. According to the analysis or examination of their study, demonstrated the potential of renewable energies in lowering carbon emissions by demonstrating the lower carbon footprint of renewable technologies compared to technologies based on fossil fuels. They discovered that renewable energies had a favorable effect on reducing carbon emissions in seven different locations (See Hertwich et al. 2015; Waris et al. 2023; Khan et al. 2021; Muhammad and Khan 2021 for similar results). In a similar vein, Namahoro and Wu (2023) investigated how economic growth and renewable sources interact to affect CO2 emissions in leading solar energy producers. They discovered that the combination of growth and renewables could affect CO2 emissions in both favorable and unfavorable ways. In order to move toward a more environmentally friendly future, Kilci (2022) underlined the significance of producing power with a small carbon impact. Rezaei Sadr et al. (2022) conducted research on how the Paris Agreement, the use of fossil fuels, and net energy imports affected CO2 emissions in three West European nations. They discovered that increasing the use of renewable energies had a detrimental effect on CO2 emissions. The study by Ganegodage et al. (2021) analyzed how grid-connected photovoltaic (PV) technology might reduce carbon emissions from buildings in Sri Lanka. They emphasized the country’s low adoption of grid-tied PV technology and the need to emphasize its significance in lowering carbon emissions. Raza et al. (2020) examined the disproportionate environmental impacts of Pakistan’s renewable energy use. Using the nonlinear-ARDL, they found that switching to renewable sources had a nonlinear effect on CO2. Gnangoin et al. (2022) examined how environmental taxes and regulations affected the deployment of renewable sources in advanced economies. They found that eco-friendly tools advocate for renewable energies and promote the swapping of dirty fuels for greener alternatives. Bashir et al. (2022) examined the correlation between renewable and non-REC, GDP growth, and carbon dioxide emissions in nations with expanding economies. They discovered that if renewable energy sources were used in tandem with human capital, carbon dioxide emissions might be lowered. According to research by Mongo et al. (2021), energy consumption has a statistically significant impact on CO2 emissions in Africa. They emphasized the potential advantages of increasing the proportion of renewable energies in the energy mix, such as a decrease in GHG emissions and an increase in GDP. Kahia et al. (2020) looked at how REN and economic development work together to achieve environmental sustainability in Saudi Arabia. They discovered that the REC has no effect on slowing down environmental deterioration indicators and that the combined effect of REC and economic growth on CO2 emissions is negligible.

An extensive analysis of the prior literature is presented. Numerous analyses have looked at how different variables affect the emissions of CO2. Still, there is little research available that looks at how different approaches, such as eco-innovation, REC, and environmental taxes, play a role in the development of policies to reduce carbon dioxide (CO2) emissions. This study aims to be the first of its kind by analyzing the effect of different measures on reducing CO2 emissions in the 21 OECD countries and addressing the climate change challenge. The findings of this study can be used as a basis for policy decisions by the many groups working to achieve Goal No-13 of the UN Sustainable Development Agenda.

## Research methodology and data identification

### Theoretical justification and elaboration of the econometric model

The objective of this research is to investigate the impact of green innovation, renewable energy consumption (REC), and environmental tax (ETAX) within the framework of OECD countries. The theoretical basis of this study is based on the theory of sustainable development. This theory offers a broader contextual framework and underscores the importance of achieving a sustainable equilibrium between economic advancement and the welfare of society and the environment over an extended period. The utilization of the innovation hypothesis is also employed in the research, which examines the impact of technological advancements and the dissemination of environmentally sustainable technologies on energy habits and, subsequently, environmental quality. Theories pertaining to environmental policy and regulation provide valuable insights into the development and implementation of policies that seek to foster sustainability and mitigate the rate of environmental deterioration. The resource-based view (RBV) theory elucidates the impact of business resources and strategies on the environmental practices of said businesses, while the ecological modernization theory provides valuable perspectives on the potential transition of industries and societies towards more environmentally sustainable practices. The application of political economics theory proves valuable in comprehending the intricate political dynamics inherent in the formulation of environmental policy decisions. The researcher’s decision to ground the study in a diverse theoretical landscape has provided a solid foundation for understanding the intricate network of factors that impact environmental quality in OECD countries. The following model explains the specification of the study as under:

1 $${CO}_{2,i,t}={\lambda}_1{lnEI}_{i,t}+{\lambda}_2{lnGDP}_{i,t}+{\lambda}_3{lnREC}_{i,t}+{\varepsilon}_{i,t}$$

where CO2 and GDP represent carbon emissions and gross domestic product, respectively. Moreover, EI shows environmental innovation proxied by the development of eco-friendly technologies as % of all technologies, and REC measured as a percentage of total final energy consumption. Furthermore, environmental tax is also adjusted as an explanatory variable in the model. The expended model is as given:

2 $$CO{2}_{2,i,t}={\lambda}_1{lnEI}_{i,t}+{\lambda}_2{lnGDP}_{i,t}+{\lambda}_3{lnREC}_{i,t}+{\lambda}_4{lnETAX}_{i,t}+{\varepsilon}_{i,t}$$

where ETAX represents environmentally related taxes, % GDP. Eco-innovation is a strategy that businesses use to increase their economic efficiency in the manufacturing process. The term “eco-innovation” refers to the practice of minimizing negative effects on the environment while simultaneously making the most of available resources. Therefore, eco-innovation is predicted to negatively affect carbon dioxide emissions $\left({\lambda}_1=\frac{\partial lnCCO2}{\partial lnei}<0\right)$. The environment is under constant threat from the world’s tremendous expansion in output, and billions of people’s lives are in danger as a result. Consistent growth of economic output increased GDP, which in turn increased demand for energy leading to a rise in CO2 emissions (Mitić et al. 2017; Tiwari 2011; Ameyaw and Yao 2018). In addition, rising levels of production are linked to a greater ecological footprint due to the ongoing depletion of natural resources. This phenomenon may be explained by the fact that natural resources are becoming increasingly scarce. As a consequence of this, we believe that both the gross domestic product and the value added to industry will contribute to a reduction in CO2 emissions $\left({\lambda}_2=\frac{\partial lnCO2}{\partial lngdp}>0\ \right)$.

Equally crucial in the effort to cope up with the problem of GHG is the REC. The theoretical foundation for the inverse link between REC and GHG emissions is the fact that REC are eco-friendly and sustainable, which can meet the demand in the present and future (Waheed et al. 2018). The foregoing reasons suggest that switching to renewable energy sources will reduce CO2 emissions $\left({\lambda}_3=\frac{\partial CO2}{\partial lnrec}<0\right)$. Similarly, The environmental tax (ETAX) is crucial to cutting down on pollution. Environmental taxes have been shown to negatively effect on CO2 emissions, with the underlying theory being that such taxes discourage the use of fossil fuels by corporations and governments. Hence, ETAX is theoretically expected to negatively affect the emissions of CO2 $\left({\lambda}_3=\frac{\partial CO2}{\partial lnrec}<0\right)$.

The process of selecting the variables for this inquiry has been conducted with careful planning, aiming to facilitate a comprehensive understanding of the research topic. The existing body of research gives substantial support for these alternatives. Numerous studies have demonstrated that the implementation of green innovation exerts a positive impact on the preservation of the environment. Extensive research has consistently shown the significance of this characteristic. Several studies, including Ali et al. (2021), Safi et al. (2022), Chi et al. (2021), and Ameyaw and Yao (2018), have emphasized the importance of technological advancements in reducing emissions and optimizing resource use. The implementation of renewable energy sources has been shown to significantly reduce greenhouse gas emissions and enhance air quality in countries that have made substantial investments in these technologies (Wang et al. 2020; Li et al. 2020; Kirikkaleli et al. 2021). The aforementioned conclusions were derived from studies conducted by Wang, Li, and Kirikkaleli. Previous studies conducted by Ji et al. (2018) and Khan et al. (2021) have examined the efficacy of carbon pricing systems. These studies have highlighted the significance of carbon taxes as a crucial policy tool since they have garnered considerable interest for their potential to mitigate carbon emissions. The research conducted by Ji et al. (2018) and Khan et al. (2021) has demonstrated the existence of this possibility. In summary, there exists a significant body of research that examines the variable of environmental quality, specifically focusing on the evaluation of air and water quality, biodiversity, and broader ecological metrics. Numerous studies have demonstrated the interrelationship between environmental policies and indicators of quality, as highlighted by the Intergovernmental Panel on Climate Change (IPCC) Beck and Mahony (2018). This study contributes to the establishment of the fundamental principles underlying environmental quality. These parameters, which have been informed by a substantial body of research, are positioned to provide useful insights into the correlation between eco-friendly innovation, carbon taxation, renewable energy, and the state of the environment in OECD countries.

### Data

The data on all variables are obtained from OECD (2021). Analysis of this study is based on the 21 selected OECD countries (shown in Table 6). The study uses CO2 emissions as dependent variable to capture the environmental performance. Eco innovation (EI), Renewable-energy consumption, GDP and ETAX are used as independent variables. The description, unit of measurement and sources of data are mentioned in Table 1.

**Table 1:** Data description

Variables	Description	Measure	Data source
CO2	Carbon emissions per capita	Metric tons	Global Carbon Project
EI	Eco innovation	% All technologies	OECD (2021)
REC	Renewable-energy consumption	% of total final energy consumption	OECD (2021)
GDP	GDP per capita	Current US$	OECD (2021)
ETAX	Environmentally related taxes	% GDP	OECD (2021)

The descriptive statistics are given in Table 2.

**Table 2:** Descriptive statistics

Model variables	Obs:	Mean:	Std.Dev:	Min	Max
lnco2	315	5.211	1.331	3.266	8.721
Lngdp	315	27.473	1.197	25.441	30.625
Lnrec	315	2.715	.74	.441	4.113
Lnei	315	.789	.386	-.33	1.579
Lnetax	315	2.419	.257	1.633	3.25

### Analytical techniques

This study employed instrumental variable methodology and is based on the following analytical techniques for estimating the relationship between the variables in models.

#### Generalized method of moments (GMM)

To assess how various factors affect a country’s energy efficiency, research scholars frequently employ the GMM methodology. It is common practice to employ GMM in the setting of OECD countries to investigate the connection between GDP, EI, and ETAX and CO2 emissions. The GMM system estimator approach thrives in the analysis of panel data. It enables the researcher to account for variations in the link between the variables of interest that may result from factors that are not directly observable. More depth can be derived from the study of the variables when the researcher is able to distinguish between instantaneous and delayed impacts. In this setting, the GMM method could be used to examine a number of hypotheses. The topic of whether or not increased eco-innovation can be achieved through GDP growth is an important one. Several GMM-based studies have found that eco-innovation can help cut down on CO2 emissions while boosting the economy. This is because eco-innovation may promote the creation of greener, more efficient technology, which in turn can help cut down on emissions and boost the economy. These studies also demonstrate that GDP expansion dampens the effect of eco-innovation on CO2 emissions. GMM could also be used to test the premise that if eco-innovation is increased, greenhouse gas emissions will decrease. This may seem like a no-brainer but changes in consumer behavior and technical advancements are only two examples of the many factors that might affect the correlation between eco-innovation and lower emissions. Typically, econometric models, including variables pertaining to GDP growth, eco-innovation, and emissions, are used when applying GMM to these issues. The model may account for confounding variables, such as economic development, population density, and policy choices, which may have an impact on the causal connection. The possible effects of high GDP growth and efficiency measures on CO2 emissions can be better understood with the use of GMM analysis. Recent research, for instance, has shown that GDP growth can contribute to increases in eco-innovation, which in turn can lead to significant decreases in emissions. Nevertheless, studies also show that these connections can be intricate, so government measures are required to guarantee that increases in GDP growth and eco-innovation translate into substantial cuts in CO2 emissions. When considering these important questions in the framework of global GDP growth and carbon emissions, the GMM method emerges as a potent technique.

#### Two-stage least square regression (2SLS)

To deal with endogeneity problems, many empirical investigations employ the 2SLS econometric methodology. Using the 2SLS method, we establish unbiased estimates of the causative links between eco-innovation, GDP growth, REC, environmental taxes, and CO2 emissions for selected 21 OECD member nations. The 2SLS method is a two-step estimation procedure. In the initial phase, we choose a group of instrumental variables to use in resolving the endogeneity problem. In the beginning, these instrumental factors need to have associations with the endogenous variables, and secondly, they need to be independent of the error term. It is found that EI, GDP growth and REC were all positively correlated with instrumental variables. As an instrumental variable, we utilized the lagging value of the ETAX. Stage two involved employing instrumental factors as indicators of endogenous variables to derive objective measures of correlation and causation.

The present analysis employed robust methodologies (such as GMM, 2SLS, and quantile regression), specifically developed to effectively tackle the challenges arising from endogeneity and cross-panel correlations. GMM is a method that effectively addresses endogeneity challenges by employing lagged variables as instruments. This methodology facilitates the collection of dynamic interactions and mitigates bias in parameter estimations. In contrast, quantile regression allows for a more nuanced examination of the influence of factors at various quantiles of the distribution, hence offering valuable insights into potential heterogeneity and distributional consequences. The incorporation of instrumental variables and the application of a structural equation modeling approach are two methods via which the two-stage least squares technique enhances the handling of endogeneity. This assertion holds particular significance within the framework of policy variables, specifically carbon taxes. By integrating these research methodologies, the study not only acknowledges the intricate interplay among the variables but also provides a comprehensive and dependable examination of the relationship between green innovation, renewable energy, carbon taxes, and environmental quality. Moreover, the study effectively tackles the issues of endogeneity and cross-panel correlations. The integration of both of these research methodologies enables the achievement of this outcome. The utilization of this particular methodological framework enhances the veracity and comprehensiveness of the research results, so yielding a more intricate understanding of the intricacies associated with policy outcomes and environmental ramifications inside the member nations comprising the OECD. Hence, this research shows how crucial it is to address endogeneity issues that can bias estimates by employing proper econometric approaches like the 2SLS method in empirical investigations if accurate estimates of causal links between variables are to be obtained. Such methods can inform policy decisions by revealing which policy interventions work best at bringing about the intended results.

## Results and discussions

This study employs the generalized method of moments (GMM) and instrumental variables (2SLS) regression approaches to estimate models 1 and 2. The results suggest that eco-innovation, GDP, REC, and ETAX are important variables affecting CO2 emissions in OECD countries. Increases in eco-innovation and REC have been found to reduce OECD countries’ CO2 emissions, as indicated by their adverse and substantial statistical coefficients. CO2 emissions, on the other hand, are positively correlated with GDP. To be specific long-run elasticity of GDP, REC, and EI are −0.496%, 0.475%, and 0.890%, respectively (model 1). Next, we introduce environmental tax as an explanatory variable in the model. With the inclusion of ETAX, the performance of the model is improved. Especially the long-run elasticity of gross domestic product, EI, REC, and ETAX are −0.706%, 0.458%, 0.861%, and 0.134%, respectively (see model 2). In addition, Table 2 represents the following results. Firstly, there is an adverse correlation between economic innovation and carbon dioxide emissions. In the long term, eco-innovation reduces CO2 emissions by an average of 0.67 percent. Furthermore, economic innovation causes a short-term 0.68% decrease in CO2 emissions. Because it helps move the economy towards a greener energy source, economic innovation helps to reduce CO2 emissions and support environmental performance. In order to minimize greenhouse gas emissions, a country can implement policies to strengthen innovation without placing heavy taxes on businesses may reduce CO2 reduction. The aforementioned results corroborate those of Guedie et al. (2022) and Töbelmann and Wendler (2020). Secondly, there is a negative and statistically significant impact of ETAX on CO2 emissions in the selected economies. This evidence supports the conclusion that ETAX is a useful policy instrument for cutting CO2 emissions in advanced economies. The results indicate that higher ETAX on emissions can effectively deter polluting practices and hasten the development of new clean technologies. Finding an acceptable compromise between economic development and CO2 emissions is the subject of this study, which also serves as a call to responsibility. The aforementioned results corroborate those of Khan et al. (2021), Muhammad et al. (2020), and Haug and Ucal (2019). Thirdly, there is a correlation between GDP and consumption-based carbon emissions that needs to be clarified. Long-term GDP growth is the primary driver of CO2 emissions. Meanwhile, GDP is responsible for a short-term increase in CO2 emissions as well. Increases in gross domestic product (GDP) have a beneficial influence on carbon dioxide (CO2) emissions because rising economic activity necessitates increased energy use. The aforementioned results corroborate those of (Ma et al. 2022b; Ding et al. 2021).

Fourthly, the use of renewable energy sources and emissions brought on by human consumption are inversely related. In the long run, using renewable energy sources results in a reduction of 0.041% in carbon dioxide emissions. In addition, using renewable energy sources results in a temporary reduction in CO2 emissions. These results support the findings of York and McGee (2017); Yuan et al. (2022), Hanif et al. (2019), and Waris et al. (2023). Considering that renewable energy technology makes use of clean and more environmentally friendly forms of energy that meet the demands of the present and the future, it is the primary means by which CO2 emissions can be reduced.

Finally, there is a short-term indication of disequilibrium, which is rectified by the 92% speed (model 2). The error correction term showed a statistically significant coefficient with adverse impact. This demonstrates reasonable convergence to the long-run equilibrium in less than a year, eliminating the gap between CO2 emissions and its repressors Tables 3, 4 and 5.

**Table 3:** Generalized method of moments’ estimates

Variables	Model 1 coefficient	Model 2 coefficient
Lngdp	0.696***	0.706***
	[0.112]	[0.121]
Lnrec	−0.475***	−0.458***
	[0.136]	[0.166]
Lnei	−0.890**	−0.861***
	[0.372]	[0.408]
Lntax	---	−0.134
		[0.514]
Constant	−11.909***	−11.923
	[3.587]	[3.497]
Mean dependent var	5.211	5.211
Number of obs	315.000	315.000
SD dependent var	1.331	1.331
Chi-square	620.268	653.998

***p<0.01, **p<0.05, *p<0.1

**Table 4:** Instrumental variables (2SLS) regression estimates

Variables	Model 1 (Coefficients)	Model 2 (Coefficients)
Lngdp	0.440***	0.465***
	[0.139]	[0.128]
Lnrec	−1.019***	−0.992***
	[0.263]	[0.248
Lnei	−0.670***	−0.678***
	[0.100]	[0.099
Lntax	---	0.301**
		[0.143
Constant	−4.260	−5.672
	[4.251]	[3.763]
Mean dependent var	5.211	5.211
R-squared	0.831	0.840
Chi-square	1204.921	1304.415
Prob > chi2	0.000	0.000

***p<0.01, **p<0.05, *p<0.1

**Table 5:** Estimates of quantile regression–MMQR

Variable	Quantiles			
	Q_0.25_	Q_0.50_	Q_0.75_	Q_0.90_
Lngdp	0.844***	0.829***	0.820***	0.811***
	(0.130)	(0.156)	(0.216)	(0.285)
Lnrec	−0.457***	−0.409**	−0.380	−0.352
	(0.172)	(0.206)	(0.285)	(0.377)
Lnei	−0.050***	−0.512***	−0.396***	−0.734***
	(0.116)	(0.124)	(0.027)	(0.108)
Lntax	−0.275	−0.326	−0.358	−0.389
	(0.368)	(0.441)	(0.611)	(0.808)
Drift	−18.055***	−15.986***	−14.714***	−13.468
	(3.956)	(4.721)	(6.528)	(8.631)

Estimation results are reported by the MMQR method. *** ,** and * 1%, 5% and 10% levels of statistical significance, respectively. The standard errors are in parentheses

This study further uses MM quantile regression approach to check the robustness of variables and model. The results show that GDP positively affect CO2 emissions at all quantiles. On the contrary, eco-innovation, ETAX, and REC negatively affect CO2 emissions at all quantiles. However, the impact of GDP, EI, ETAX, and REC on CO2 emissions varies across different quantiles, suggesting heterogeneity in the relationships. The coefficient of GDP is increasing with successive quantiles, which suggests that countries with lower CO2 emissions have a stronger positive relationship between GDP and CO2 emissions, possibly due to a greater reliance on biomass-based technologies. Similarly, the coefficient of EI is increasing with a successive quantile, which suggests that in countries with lower CO2 emissions, there is a strong impact of eco-innovation on environmental performance.

### Discussion

The study's findings provide insights into several noteworthy elements that contribute to carbon dioxide (CO2) emissions in countries belonging to the Organization for Economic Cooperation and Development (OECD). Significantly, eco-innovation, the utilization of renewable energy (REC), and the implementation of environmental taxes (ETAX) exhibit negative coefficients, indicating their significant contribution to the mitigation of carbon emissions. The results of this study align with the existing theoretical frameworks that have been previously created. To commence, the observation that eco-innovation exhibits a negative coefficient underscores its substantial contribution to mitigating human impact on the environment. The theoretical underpinning of this conclusion is based on the notion of eco-innovation, which prioritizes the development and implementation of environmentally sustainable technologies and activities. This aligns with the theory of the resource-based view (RBV), which suggests that a company’s strengths, such as its ability to sustainably innovate, can positively impact the company’s overall environmental performance. Moreover, the negative coefficient for REC provides support for the proposition that the adoption of renewable energy sources can effectively reduce carbon dioxide emissions. This aligns with the concept of environmental economics, which places focus on the favorable environmental externalities linked to the utilization of renewable energy sources. By reducing reliance on fossil fuels and mitigating greenhouse gas emissions, the adoption of renewable energy contributes to these positive outcomes. Additional support for the validity of the theoretical framework underlying market-based environmental policy is provided by the presence of a negative coefficient associated with environmental taxes, commonly referred to as ETAX. The proposition posits that the implementation of elevated environmental taxes serves as a catalyst for both corporations and individuals to reduce their emissions, hence aligning with the concept of carbon pricing. This approach effectively incorporates the societal burden of pollution. The rationale behind this phenomenon lies in the fact that the imposition of elevated environmental taxes results in increased costs for both enterprises and individuals engaged in polluting activities.

The implications of these findings have significant importance, not just for policymakers in OECD countries, but also for firms operating within such countries. This information can serve as a valuable resource for policymakers in the development of environmental regulations and incentives that exhibit more efficiency. The presence of negative coefficients in eco-innovation, REC, and ETAX underscores the need of establishing a conducive climate for fostering green innovation and the uptake of renewable energy sources. Additionally, leveraging taxes as a means to incentivize behavioral modifications becomes crucial in this context. Furthermore, the business sector can leverage the competitive advantages provided by eco-innovation and renewable energy sources, leading to practical implementations in various business contexts. Business entities possess the opportunity to adopt ecologically sustainable business practices and technology, exploit renewable energy credits (REC), and take into account the tax consequences associated with their emissions. Furthermore, these findings underscore the importance of upholding a harmonious equilibrium between economic growth and environmental conservation throughout periods of economic expansion. Hence, it is imperative to employ strategies that facilitate the decoupling of GDP development from the escalation of CO2 emissions. In conclusion, the present study underscores the significance of adopting a comprehensive strategy in mitigating carbon dioxide emissions. In order to establish a basis for transitioning towards a future that is both ecologically responsible and prosperous, it is imperative to integrate eco-innovation, renewable energy, environmental taxes, and sustainable economic development into the approach.

## Conclusions and policy implications

### Conclusion

In conclusion, there is a great deal of depth and complexity in the relationship between EI, ETAX, REC, and ECO2 emissions. This research employed (2SLS) method, which is helpful for examining the effects of GDP while accounting for confounding variables. This study found a negative and statistically significant relationship between CO2 emissions and EI, GDP growth, and REC and a favorable and statistically significant relationship between CO2 emissions and ET. These results suggest that ET legislation, along with strategies that encourage EI, REC, and sustainable economic growth, can help OECD countries reduce their CO2 emissions. Based on the selected 21 OECD study, this study concludes that policy interventions are required to fully realize the potential benefits of GDP growth for improving energy effectiveness and decreasing greenhouse gases. Some examples of such interventions are carbon pricing mechanisms to internalize the costs of emissions or legislation to encourage the development of goods and innovations with lower emissions. Overall, our results highlight the significance of studying the issue of lowering carbon emissions, including the complex interactions between GDP growth, eco-innovation, REC, and other issues like technology and policies. While this global problem does not have a simple answer, it can be addressed through sustained study and legislative changes that lead to a more sustainable future.

### Policy recommendations

The findings of the study led us to propose several policy actions designed to cut emissions while enhancing economic growth. Among our suggestions are as follows: (a) setting a system for carbon pricing: Policy interventions like carbon pricing have been shown to be beneficial for reducing emissions. Businesses and individuals can be incentivized to minimize their carbon footprint by internalizing the cost of doing so, which can be achieved through taxation. (b) Subsidizing technological advancements: Innovative technologies that help minimize carbon emissions can be developed, implemented, and adopted with government support. More extensive utilization of environmentally friendly technologies may result from the incentives provided by these programs. (c) Boosting initiatives to reduce energy waste: Incentives for businesses and people to use energy-saving practices can also come from government policy. Energy-efficient equipment might be subsidized through tax breaks, and businesses may be encouraged to do energy audits to find ways to cut energy use. (d) Sustainable global trade can be encouraged by governments setting environmental criteria for imported products and services and providing encouragement to companies that implement low-carbon practices throughout their supply chains. (d) The importance of funding R&D efforts: Government spending on R&D can help us comprehend more fully the interconnected nature of the economy, energy consumption, and carbon emissions, leading to the creation of novel, environmentally friendly technologies and programs. By adopting these policy suggestions, OECD countries may boost their economies while cutting carbon emissions, creating an environmentally friendly future for everyone.

### Limitations and future research direction

This study is limited to analyzing the effects of environmental improvement strategies on CO2 emissions of selected OECD countries. In the future, studies may be carried out to include those factors that accelerate CO2 emissions. These factors may include trade flows, urbanization, etc. Moreover, we restricted our analysis to selected OECD countries. The findings of this study may be extended to other groups such as G7, G20, and the European Union etc.

## Data Availability

Data from the corresponding author will be available upon reasonable request.

## Appendix

**Table 6:** List of 21 OECD Countries

S. No	Countries	CO2 emissions (million tons of GHG)
1	USA	6297.62
2	Japan	1270.21
3	Germany	873.6
4	Canada	762.12
5	Australia	581.97
6	Turkey	579.19
7	UK	463.74
8	France	450.39
9	Italy	417.56
10	Spain	349.77
11	Netherlands	221.9
12	Belgium	130.56
13	Greece	91.86
14	Austria	85.39
15	Finland	74.8
16	Norway	71.01
17	Portugal	69.55
18	Hungary	67.16
19	Sweden	64.59
20	Denmark	49.17
21	Switzerland	47.91
